# Supplementary material for: Optimal minimal residual disease threshold in pediatric acute myeloid leukemia: A retrospective cohort study based on the TARGET database
Source: PLoS Med. 2026 May 8;23(5):e1005088. doi: 10.1371/journal.pmed.1005088 (PMC13155632; doi:10.1371/journal.pmed.1005088)
Supplement: S1 Code — (ZIP) [file pmed.1005088.s002.zip › S2 code/PROJ8_15_tbl1/PROJ8_15_tbl1_seg2_3.pdf]

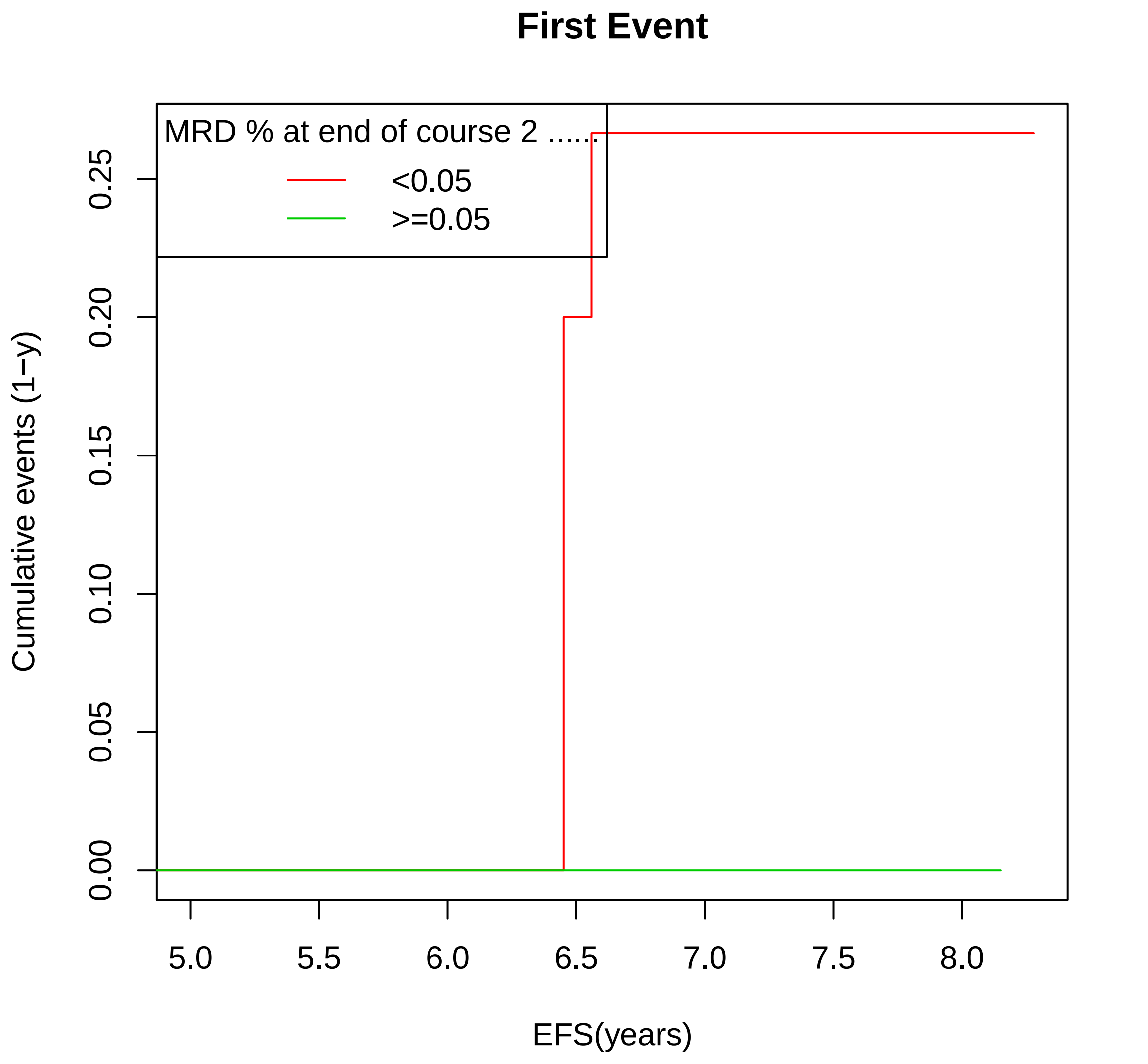

First Event
MRD % at end of course 2 ......
<0.05
>=0.05
Cumulative events (1−y)
0.00
0.05
0.10
0.15
0.20
0.25
5.0
5.5
6.0
6.5
7.0
7.5
8.0
EFS(years)
